# Suicide in Muslim‐Majority Countries Within the WHO African Region: A Narrative Review of Epidemiology and SocioCultural Factors

**DOI:** 10.1002/puh2.70326

**Published:** 2026-07-28

**Authors:** S. M. Yasir Arafat, Sadeed Hossain, David Lester, Ahmed N. Ramadan

**Affiliations:** ^1^ Department of Public and Community Health Faculty of Medicine and Health Sciences Frontier University Garowe Garowe Somalia; ^2^ Biomedical Research Foundation Dhaka Bangladesh; ^3^ Department of Psychiatry Bangladesh Specialized Hospital Dhaka Bangladesh; ^4^ Stockton University Galloway New Jersey USA; ^5^ Neuropsychiatry Department, Faculty of Medicine Menoufia University Shibin El‐Kom Egypt

**Keywords:** Africa, epidemiology, Islamic, Muslim‐majority, suicidal behaviour, suicide

## Abstract

**Background:**

Although suicide research is extensive in many countries, Muslim‐majority countries in Africa represent a unique ‘blind spot’ as a result of a complex interplay of sociocultural stigma, legal criminalization and religious prohibition.

**Objective:**

This article describes suicide in Muslim‐majority countries within the World Health Organization (WHO) African region (AFR).

**Methods:**

We assessed estimates on suicide in eleven Muslim‐majority countries (Algeria, Burkina Faso, Chad, Comoros, Gambia, Guinea, Mali, Mauritania, Niger, Senegal and Sierra Leone) located within the WHO AFR, focusing on available epidemiological patterns, relevant sociocultural, religious, legal aspects and data‐quality considerations.

**Results:**

Suicide rates varied widely across the region. Algeria reported the lowest age‐standardized rate (2.3 per 100,000), whereas Burkina Faso had the highest (15.4 per 100,000). Other countries showed moderate rates: Chad 10.7, Comoros 8.9, Gambia 8.3, Guinea 8.2, Mali 8.3, Mauritania 4.9, Niger 8.9, Senegal 11.6 and Sierra Leone 9.7 (per 100,000). Male suicide rates consistently exceeded female rates across all countries.

**Conclusion:**

Suicide in African Muslim‐majority countries results from complex interactions between individual, social, economic and structural factors. Prevention requires integrated strategies that combine evidence‐based mental health care with culture and religion sensitive approaches, supported by legal reform and strengthened community engagement.

## Graphical abstract

We assessed estimates on suicide in eleven Muslim‐majority countries located within the WHO African region, focusing on available epidemiological patterns and discussing relevant sociocultural, religious, legal and data‐quality considerations. Suicide rates varied widely across the region. Algeria reported the lowest age‐standardized rate, whereas Burkina Faso had the highest.

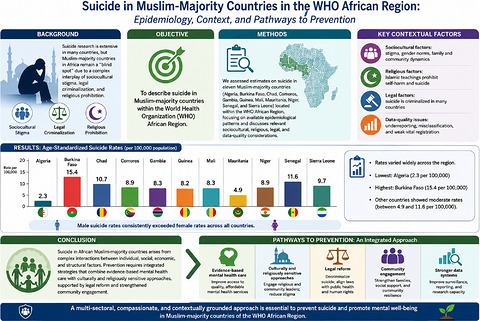

## 1 Introduction

### 1.1 Suicide: Global View

Suicide is recognized as an important and growing public health concern in the world, and the prevention of suicide is one of the major challenges across the world [1]. It is one of the leading causes of death worldwide, as every year, more than 720,000 people die by suicide. However, this estimated toll relies on the quality of mortality data, which is poor for most countries in the world [1]. Globally, around 73% of all suicides worldwide occur in low‐ and middle‐income countries (LMICs). These data underscore the complex interplay between population size, socio‐economic context and suicide mortality patterns across income groups.

### 1.2 Suicide in Africa

Africa is the world's largest and second most populous continent with a population of more than one and a half billion people [2]. Africa is characterized by a substantial diversity in culture, ethnicity, religion, language and socio‐economic conditions. The continent comprises 54 countries with a wide variation in levels of development, urbanization, political stability and health system capacity [3]. However, for public health governance and reporting purposes, the World Health Organization (WHO) does not categorize all 54 African countries within a single administrative region. The WHO African region (AFR) consists of 47 Member States, whereas several and Horn of Africa and northern countries are administratively assigned to the Eastern Mediterranean Region [4]. As a result of this, WHO reports that focus on the AFR primarily analyse data derived from these 47 AFR Member States rather than the entire continent.

### 1.3 Suicide in Muslim‐Majority Countries of WHO AFR

Suicide is a significant but often under‐recognized public health concern in Africa. The latest WHO estimate indicates that WHO AFR has the highest age‐standardized suicide rate (ASR) (11.5 per 100,000 per year) compared to the global rate of 8.9 per 100,000 [1]. Furthermore, in many countries, the true burden of suicide is masked due to the weak civil registration, social stigma and criminalization. In some African countries, suicide attempts and suicide deaths are not consistently documented because of limited surveillance systems and sociocultural barriers to reporting [5, 6]. A recent analysis identified that, among Muslim regions, sub‐Saharan African countries had the highest suicide rate, which was even higher than the global average [7]. Focusing on the WHO AFR countries allows for a more context‐specific understanding of suicide epidemiology in predominantly Muslim populations within sub‐Saharan Africa, while maintaining alignment with WHO regional reporting structures. However, it is important to note that the data quality for all included countries is categorized as ‘very low’ according to WHO assessments [1]. Consequently, suicide mortality estimates for these settings should be interpreted with caution, as under‐reporting, limited civil registration systems and sociocultural stigma surrounding suicide may affect accuracy.

Despite these limitations, available estimates provide valuable preliminary insight into the magnitude and distribution of the suicide burden across Muslim‐majority countries within the WHO AFR. This article examines suicide in eleven African Muslim‐majority countries (Algeria, Burkina Faso, Chad, Comoros, Gambia, Guinea, Mali, Mauritania, Niger, Senegal and Sierra Leone). Specifically, it focuses on the available epidemiological patterns, relevant sociocultural, religious, legal aspects and data‐quality considerations.

## 2 Methods

In this study, secondary data from different internationally recognized sources that are openly available were collected. One author extracted data, and another verified. These sources are highlighted below or later in the methods section.

### 2.1 List of Countries and Populations

The list of countries was collected from previously published articles [8, 9] and the latest WHO report [1]. We considered the countries located in the WHO AFR having >50% Muslim populations of the total. It included a list of 11 countries (Algeria, Burkina Faso, Chad, Comoros, Gambia, Guinea, Mali, Mauritania, Niger, Senegal and Sierra Leone).

We collected the total populations (in millions) and proportion of Muslims of an individual country from the World Population Review [2, 10].

### 2.2 Suicide Rate, Data Quality and Income

Suicide rates (in total population, males, females), ASRs, for all ages (per 100,000), number of suicides, income distribution of the country, data quality and distribution of the WHO region were extracted from the WHO reports that includes the suicide estimates for 2021, 2019, 2016, and 2012 [1, 11, 12, 13].

The economic status of the included countries was classified according to the WHO [1]. It should be noted that the reported suicide rates differ in the WHO and in the World Bank and other data sources. We have relied on WHO rates for the present paper. In addition, some data sources report crude suicide rates, whereas others report ASR.

### 2.3 Human Development Index (HDI)

HDI was extracted from the Global Human Development Indicators of 2025 [14]. HDI values were extracted for each country, ranging from 0.41 for Chad to 0.76 for Algeria (Table 1).

**TABLE 1:** Suicide rate and socio‐economic characteristics of Muslim‐majority countries in the WHO African region [1, 2, 8, 9, 10, 14].

SN	Country	Data quality	Income group	Total population, 2026 (million)	Total Muslim population, 2026 (million)	Percentage of Muslim population	Sex	Number of suicides	Age‐standardized suicide rate (per 100,000)	HDI
1	Algeria	Very low	UMI	48.02	43.73	97.9	Both	976	2.3	0.76
							Female	292	1.4	
							Male	684	3.2	
2	Burkina Faso	Very low	LI	24.6	13.51	62.7	Both	1869	15.4	0.45
							Female	443	6.7	
							Male	1426	26.2	
3	Chad	Very low	LI	21.56	9.18	55.1	Both	929	10.7	0.41
							Female	269	6.4	
							Male	660	15.4	
4	Comoros	Very low	LMI	0.89	0.80	98.3	Both	49	8.9	0.60
							Female	18	6.4	
							Male	31	11.5	
5	Gambia	Very low	LI	2.88	2.28	95.3	Both	124	8.3	0.52
							Female	40	5.4	
							Male	84	11.4	
6	Guinea	Very low	LMI	15.44	10.56	84.6	Both	661	8.2	0.50
							Female	246	5.7	
							Male	415	11.3	
7	Mali	Very low	LI	25.93	17.50	94.6	Both	931	8.3	0.42
							Female	309	5.4	
							Male	621	11.4	
8	Mauritania	Very low	LMI	5.46	4.15	99.1	Both	128	4.9	0.56
							Female	42	3.1	
							Male	87	6.9	
9	Niger	Very low	LI	28.81	21.10	98.3	Both	1097	8.9	0.42
							Female	357	5.8	
							Male	740	12.1	
10	Senegal	Very low	LMI	19.36	17.42	96.6	Both	1164	11.6	0.53
							Female	252	4.9	
							Male	912	18.4	
11	Sierra Leone	Very low	LI	8.99	6.06	78.5	Both	497	9.7	0.46
							Female	182	6.9	
							Male	315	12.9	

Abbreviations: HDI, Human Development Index; LI, low‐income countries; LMI, low middle‐income countries; UMI, upper middle‐income countries.

## 3 Results

### 3.1 Epidemiology of Suicide Across Countries

As shown in Table 1, a total of 11 Muslim‐majority countries within the WHO AFR were included in the analysis. All countries were categorized as having very low data quality according to the WHO classification [1]. Of these, six were low‐income (LI), four were lower middle‐income (LMI), and one was upper middle‐income (UMI) [1].

The number of suicides (both sexes combined) ranged from 49 in Comoros to 1869 in Burkina Faso [1]. ASR varied substantially across countries, from 2.3 per 100,000 population in Algeria to 15.4 per 100,000 in Burkina Faso [1]. Relatively elevated ASRs were also observed in Senegal (11.6), Chad (10.7), Sierra Leone (9.7), and Niger and Comoros (8.9 each). In contrast, Mauritania (4.9) and Algeria (2.3) had the lowest rates within the region [1].

### 3.2 Sex‐Specific Patterns

Across all included countries, males consistently exhibited higher suicide rates and higher absolute numbers than females. Male ASRs ranged from 3.2 per 100,000 in Algeria to 26.2 per 100,000 in Burkina Faso, whereas female ASRs ranged from 1.4 per 100,000 in Algeria to 6.9 per 100,000 in Sierra Leone [1].

The largest sex disparity was observed in Burkina Faso, where the male ASR (26.2) was nearly four times higher than the female ASR (6.7). Similar sex differences were also evident in Senegal (18.4 vs. 4.9) and Chad (15.4 vs. 6.4) [1]. Even in countries with comparatively lower overall suicide rates, such as Algeria and Mauritania, male suicide rates remained more than double those of females.

In numbers, male suicides accounted for the majority of deaths in every country, including 1426 of 1869 deaths in Burkina Faso, 912 of 1164 in Senegal, 740 of 1097 in Niger and 684 of 976 in Algeria [1].

### 3.3 Income Group and Development Indicators

Countries classified as LI generally demonstrated moderate‐to‐high suicide rates. Burkina Faso (LI) recorded the highest ASR (15.4), followed by Chad (LI, 10.7). Among LMI countries, Senegal (11.6) exhibited one of the highest ASRs in the region [1]. Algeria, the only UMI country included, reported the lowest ASR (2.3) [1].

### 3.4 Human Development Index

HDI values ranged from 0.41 in Chad to 0.76 in Algeria [14]. Countries with HDI values below 0.50 (Burkina Faso, Chad, Mali, Niger and Sierra Leone) generally had ASRs between 8.2 and 15.4 per 100,000 population. Conversely, Algeria, with the highest HDI (0.76), reported the lowest suicide rate (2.3 per 100,000) [14]. Mauritania, despite a moderate HDI (0.56), exhibited a relatively low ASR (4.9), indicating some variability in the relationship between development indicators and suicide burden [1, 14].

### 3.5 Suicide Trend

#### 3.5.1 Algeria

**FIGURE 1:**
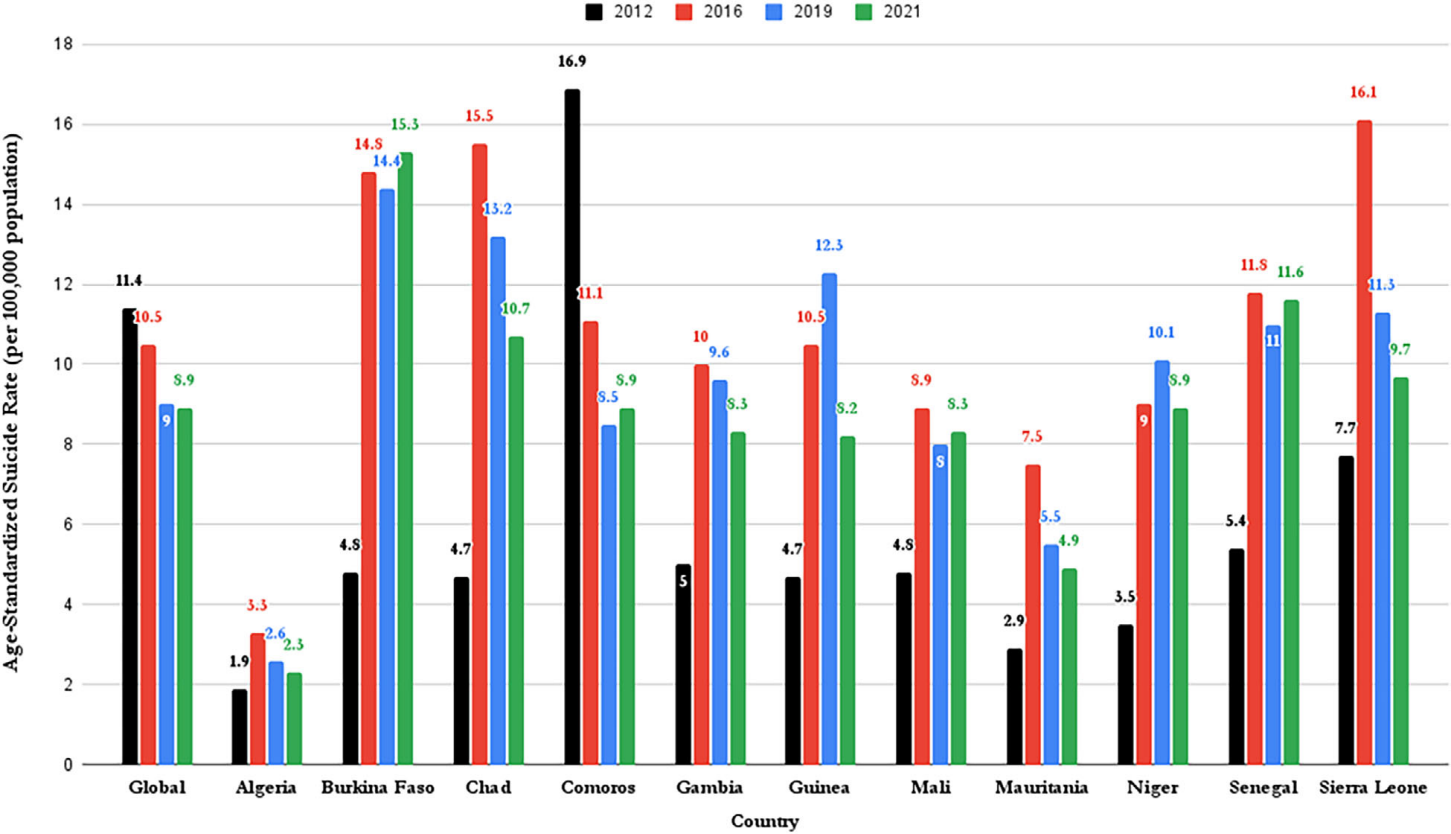
Trends in age‐standardized suicide rates (per 100,000 population) in Muslim‐majority countries of the WHO African region, 2016–2021.

Suicide rates in Algeria have remained low compared to many other countries in the WHO AFR and the global average (Figure 1). The ASR steadily decreased from 3.3 per 100,000 population in 2016 to 2.6 in 2019 and 2.3 in 2021 [1, 11, 12].

#### 3.5.2 Burkina Faso

Burkina Faso has comparatively higher suicide rates within the region. The ASR decreased slightly from 14.8 in 2016 to 14.4 in 2019, then increased to 15.3 per 100,000 in 2021, indicating general stability with small fluctuations [1, 11, 12].

#### 3.5.3 Chad

Chad showed a decline in suicide mortality over time. The ASR decreased from 15.5 in 2016 to 13.2 in 2019 and further to 10.7 per 100,000 in 2021 [1, 11, 12].

#### 3.5.4 Comoros

Comoros experienced some fluctuations in suicide rates. The ASR declined from 11.1 in 2016 to 8.5 in 2019, then slightly increased to 8.9 per 100,000 in 2021 [1, 11, 12]. Interestingly, the 2012 rate (16.9) was much higher than later years, which may be due to reporting differences rather than a real spike [13].

#### 3.5.5 Gambia

Gambia demonstrated a steady decline in suicide mortality. The ASR decreased from 10.0 in 2016 to 9.6 in 2019 and further to 8.3 per 100,000 in 2021 [1, 11, 12].

#### 3.5.6 Guinea

Although the ASR decreased overall from 10.5 in 2016 to 8.2 in 2021, there was a noticeable increase in 2019 (12.3 per 100,000) [1, 11, 12].

#### 3.5.7 Mali

Mali's suicide rate has remained relatively stable. The ASR declined slightly from 8.9 in 2016 to 8.0 in 2019 and then increased marginally to 8.3 per 100,000 in 2021 [1, 11, 12].

#### 3.5.8 Mauritania

Mauritania showed a consistent downward trend. Over the past two decades the ASR decreased from 7.5 in 2016 to 5.5 in 2019 and 4.9 per 100,000 in 2021 [1, 11, 12].

#### 3.5.9 Niger

Niger's suicide rates remained generally stable with minor fluctuations. The ASR was 9.0 in 2016, increased to 10.1 in 2019 and slightly decreased to 8.9 per 100,000 in 2021 [1, 11, 12].

#### 3.5.10 Senegal

Senegal experienced relatively stable suicide mortality. The ASR decreased from 11.8 in 2016 to 11.0 in 2019 and then increased slightly to 11.6 in 2021 [1, 11, 12]. The suicide mortality rate has shown low temporal variation over the past two decades, reaching its lowest level in 2014 (5.4 per 100,000 population) [13].

#### 3.5.11 Sierra Leone

Sierra Leone showed one of the most noticeable declines. The ASR dropped from 16.1 in 2016 to 11.3 in 2019 and further to 9.7 per 100,000 in 2021 [1, 11, 12].

## 4 Discussion

### 4.1 Cross‐Country Comparison of Suicide Epidemiology

Across the 11 Muslim‐majority countries within the WHO AFR, substantial heterogeneity in suicide rates was observed despite shared religious majority and similar regional classification.

ASR varied more than six‐fold, ranging from 2.3 in Algeria to 15.4 in Burkina Faso [1]. Comparatively high rates were also observed in Chad (10.7) and Senegal (11.6), whereas lower rates were reported in Mauritania (4.9) and Comoros (8.9) [1].

When compared with non‐Muslim‐majority countries in Africa, the Muslim‐majority countries included in this study generally exhibited lower suicide rates than the highest burden countries on the continent. In the WHO AFR, several non‐Muslim‐majority countries report markedly high ASR. Lesotho has reported an ASR as high as 36.7 per 100,000 population, whereas Eswatini has an ASR of 31.8 and South Africa reports an ASR of 21.1. These represent the three highest ASRs among countries in the AFR and are substantially higher than the highest rate observed among the Muslim‐majority countries in our sample, Burkina Faso (15.4 per 100,000 population) [1].

When stratified by income group, LI countries (e.g., Chad, Niger, Mali and Sierra Leone) demonstrated moderate‐to‐high suicide rates (8.3–9.7 per 100,000), whereas the only upper middle‐income country, Algeria, reported the lowest rate in the cohort (2.3 per 100,000) [1]. This pattern suggests a potential inverse relationship between economic development and suicide burden within this subset. A similar finding was noted in a previous study with 46 Muslim‐majority countries with the same data source, where suicide rate was inversely associated with income of the country [9].

Countries with lower HDI (<0.50), including Burkina Faso (0.45), Chad (0.41), Mali (0.42) and Sierra Leone (0.46), have higher suicide rates compared with higher HDI settings [14]. Algeria (HDI 0.76) demonstrated the lowest suicide burden, supporting a possible association between human development and suicide mortality. A similar finding was noted in a previous study with 46 Muslim‐majority countries, where suicide rate was inversely associated with the HDI score of the country [9]. However, notable exceptions exist, and additional studies are warranted to establish the association.

### 4.2 SocioCultural Factors for Suicide in African Muslim Countries

Suicide occurs within a complex interplay of individual, sociocultural, economic and structural determinants. Understanding risk factors within these settings, therefore, requires a multidimensional framework integrating clinical, cultural and structural determinants.

#### 4.2.1 Mental Health Disorders

Consistent with global evidence, psychiatric disorders remain one of the strongest risk factors for suicide. Depression, anxiety disorders, substance use disorders and post‐traumatic stress disorder (PTSD) are frequently implicated [15, 16]. In Sierra Leone and Guinea, survivors of Ebola outbreaks experience depression and anxiety, and more than 1 in 10 survivors report self‐harm or suicidal ideation [17]. However, in many African countries mental health services are under‐resourced, and treatment remains inadequate [18]. Stigma surrounding mental illness often is influenced by religious misinterpretations which increase mental distress and may further delay care‐seeking [19].

#### 4.2.2 Sociopolitical Instability and Conflict

Several Muslim‐majority countries in sub‐Saharan Africa have experienced prolonged armed conflict, insurgency, displacement and political instability [20, 21]. In Burkina Faso, conflict‐related displacement, death of loved ones and chronic insecurity of food and life have been associated with raised psychological distress among Malian refugees [22]. Exposure to violence, forced migration and chronic insecurity increases the prevalence of trauma‐related disorders and psychological distress.

#### 4.2.3 Poverty and Economic Stressors

Most Muslim‐majority countries within the WHO AFR are classified as low‐ or lower middle‐income economies [1]. Chronic poverty, unemployment and food insecurity may influence psychosocial stress [23]. Financial strain or poverty can be associated with suicidal behaviour and may be a risk factor in countries where social protection systems are weak [24].

#### 4.2.4 Gender and Sociocultural Dynamics

Marked sex differences in suicide patterns are observed, with males having higher suicide mortality rates than females in Muslim‐majority countries of Africa. Gender norms emphasizing male economic responsibility, traditional masculinity and restricted emotional expression may increase male vulnerability to suicide due to maladaptive coping and social exclusion [25]. This pattern reflected the country's sociopolitical and economic context, including the legacy of civil war, persistent poverty and limited access to healthcare services [26].

#### 4.2.5 Limited Mental Health Infrastructure

The treatment gap for mental disorders in sub‐Saharan Africa is high. Mental health services are mostly centralized in urban areas with minimal integration into primary care. There is also underfunding, a lack of trained healthcare professionals, social stigma and inadequate facilities [27]. In Chad, most modern mental health services are available only in the capital city, with very limited or no services in rural and remote areas. In addition, the country does not have fully functioning mental health legislation or a comprehensive national mental health strategy to guide service delivery. Combined with a severe shortage of trained mental health professionals, these gaps significantly limit access to care for people experiencing mental health problems [28]. In such resource‐constrained settings, individuals experiencing suicidal ideation may lack access to proper psychological or psychiatric support.

#### 4.2.6 Religion as a Protective Factor

Religion has been identified as one of the protective factors of suicidality, and intrinsic religiosity is protective against suicidal ideation and attempt [29]. Islamic teachings strongly prohibit suicide and emphasize the sanctity of life, potentially serving as a protective cultural factor. Religious participation and spiritual coping mechanisms may reduce suicide risk in some contexts [30]. However, the suicide rate is significantly higher in African Muslim countries than in others [9]. Therefore, reliance solely on spiritual approaches without integration of evidence‐based mental health care may delay professional intervention. Various studies suggest that culturally adapted models integrating psychological treatment with spiritual frameworks may be particularly relevant for Muslim‐majority settings [31]. Higher levels of religiosity have also been cited as a possible explanation for comparatively lower suicide rates observed in many Muslim populations. However, this relationship is not uniform. Some Muslim‐majority countries, including Burkina Faso, Chad and Sierra Leone, report suicide rates that exceed the global average, and all are located within the WHO AFR [32].

### 4.3 Legal Status of Suicide in African Muslim Countries

Suicide has been decriminalized in most Western countries and in many nations with low proportions of Muslim populations. In contrast, in several Muslim‐majority countries, suicidal behaviour remains legally treated as a criminal offence punishable by fines and/or imprisonment [8].

African Muslim countries, like Algeria, Morocco, Djibouti, Somalia and Sudan, follow the Islamic Shariah law, where attempting suicide is treated as a criminal offence [33]. As suicidal behaviour is punishable by law, individuals may fear arrest, legal procedures, fines or imprisonment. This fear can discourage people from seeking medical treatment or psychological support after a suicide attempt [33, 34].

Studies have found that the criminal legal status of suicide does not prevent suicidal behaviours. Instead, criminalization of suicide may increase stigma and shame, making individuals and families less willing to disclose suicide attempts or self‐harm [35, 36, 37, 38].

When suicide is treated as a criminal offence, many cases may go unreported. Families and communities may avoid informing authorities about suicide or suicide attempts because of legal harassment or social stigma. In some situations, deaths may be recorded under other causes to avoid legal complications. As a result, official statistics may not document the true number of suicides. This leads to under‐reporting and inaccurate national suicide rates, which makes it difficult for governments and public health authorities to understand the real magnitude of the problem and to plan effective prevention strategies [39].

### 4.4 Data Quality and Possible Under‐Reporting and Misclassification

Suicide research in Africa is limited due to a lack of systematic data collection. With less than 10% of African countries reporting mortality data to the WHO, official statistics are available for only 15% of the continent's total population. Much of the available published suicide data are based primarily on small studies conducted in different regions and populations. Moreover, reported suicide mortality statistics are likely to underestimate the true magnitude of the problem as religious and cultural sanctions may lead to suicide being under‐reported, misclassified or deliberately concealed [40].

Suicidal behaviour in Africa is likely under‐reported due to multiple factors like research limitations, resource constraints, sociocultural influences, religious beliefs, financial reasons and the potential misclassification of suicides as ‘undetermined’ causes of death. Negative cultural sanctions and the criminalization of suicidal behaviour in certain countries further contribute to non‐reporting [6].

The contribution of WHO AFR to global suicide literature is negligible. One recent study found that the region contributes to 1.5% of global suicide research [41]. Another study found an extreme dearth of suicide research in the African Muslim‐majority countries [42]. When comparing the Muslim‐majority countries in various regions, African countries are not featured in the leading contributors [43].

### 4.5 Suicide Prevention in WHO AFR Muslim‐Majority Countries

Among the 11 countries (Algeria, Burkina Faso, Chad, Comoros, Gambia, Guinea, Mali, Mauritania, Niger, Senegal and Sierra Leone), none has a publicly documented national suicide prevention strategy. However, Algeria is the only country currently in the process of developing such a strategy, which is not yet completed [44].

Low suicide rates among Muslims are attributed to their level of religiosity [45, 46]. According to [47], religion can lower suicidal behaviour by providing a network of social support, fostering self‐esteem, offering life goals and enhancing coping mechanisms in times of stress and crisis. Religious affiliation may be associated with a lower risk of both suicide attempts and death by suicide through multiple mechanisms, including the encouragement of social support, personal empowerment, promotion of a healthy lifestyle and adherence to life‐preserving religious principles [48]. Religious education also helps people cope with daily socio‐economic, health and cultural challenges, which can lower the risk of suicidal behaviour [49, 50, 51]. Assessing religiosity as part of routine mental health assessments may help to identify potential therapy targets and ways to strengthen life‐affirming beliefs and expectations [52].

Given the scarcity of psychiatrists in sub‐Saharan Africa, suicide prevention is often ‘task‐shifted’ to the community. Specific programmes, such as the Campus Connect model adapted for African universities, train non‐professionals—including teachers, youth leaders and elders—to identify key warning signs that may help prevent suicide [53].

### 4.6 Strengths and Limitations of the Present Study

This study provides a comprehensive overview of suicide burden, risk factors and sociocultural determinants across all African Muslim‐majority countries in the AFR, using publicly available data.

However, several limitations need to be acknowledged. Data quality was classified as very low in all included countries, reflecting under‐reporting, misclassification and incomplete civil registration. Additionally, cross‐country comparisons are limited due to the heterogeneity in surveillance, reporting standards and the limited availability of data. The study used data from secondary sources, which limits the ability to capture nuanced cultural and community‐level factors influencing suicidal behaviour. The analysis was conducted at the country level and did not include individual‐level measures of religiosity, which could be a risk of ecological fallacy. The country‐level religious affiliation could vary with individual religiosity, religious involvement or religious practice. We consider Muslim‐majority countries based on proportion of Muslim population without considering the religion of deceased, individual religiosity, religious involvement or religious practice, which could be potential areas of biases.

## 5 Conclusion

Suicide in Muslim‐majority countries located in WHO AFR is influenced by multiple factors, including religion, culture, economics and social systems. Males consistently exhibit higher suicide rates. No country discussed in the present paper currently has a fully implemented national suicide prevention strategy which highlights an urgent need for culturally sensitive, evidence‐based interventions, community‐based programmes, and legal reforms to reduce stigma and improve access to mental health care.

## Author Contributions

Conception: S. M. Yasir Arafat. Data curation: S. M. Yasir Arafat, Sadeed Hossain, and Ahmed N. Ramadan. Writing – original draft: S. M. Yasir Arafat, Sadeed Hossain, David Lester, and Ahmed N. Ramadan. Writing – review and editing: S. M. Yasir Arafat, Sadeed Hossain, and David Lester. All authors have read and approved the final version of the manuscript.

## Funding

The authors have nothing to report.

## Ethics Statement

As this review utilized publicly available data and did not involve human participants directly, ethical approval was not required.

## Conflicts of Interest

The authors declare no conflicts of interest.

## Data Availability

Data sharing is not applicable to this article as no datasets were generated or analysed during the current study.
